# Adherence of trials of operative intervention to the CONSORT statement extension for non-pharmacological treatments: a comparative before and after study

**DOI:** 10.1308/003588412X13171221592339

**Published:** 2012-09

**Authors:** R Gray, M Sullivan, DG Altman, AN Gordon-Weeks

**Affiliations:** ^1^Oxford University Hospitals NHS Trust,UK; ^2^Centre for Statistics in Medicine, Oxford,UK; ^2^University of Oxford,UK

**Keywords:** CONSORT, Randomised controlled trial, Publishing/standards, Research design

## Abstract

**INTRODUCTION:**

Use of the Consolidated Standards of Reporting Trials (CONSORT) statement has been shown to improve the reporting of randomised controlled trials and it is endorsed by leading surgical journals. The CONSORT statement for non-pharmacological treatment (CONSORT-NPT) provides specific items to aid in the reporting of trials of operative intervention. This study compares the reporting practice of trials of operative intervention published in time periods before and after publication of the CONSORT-NPT statement.

**METHODS:**

A 30-point checklist containing the salient CONSORT-NPT items was designed and the adherence of trials meeting the inclusion criteria determined independently by two authors.

**RESULTS:**

There was a significant improvement of 3.95 points in the mean CONSORT-NPT score from 2004 to 2010 (95% confidence interval: 3.61–4.29, *p*<0.001). This related specifically to items present in the original CONSORT statement rather than to CONSORT-NPT items, which remained poorly reported in 2010. The mean CONSORT-NPT score was 17.5 (standard deviation [SD]: 4.5) for trials published in CONSORT endorsing journals compared with 15.6 (SD: 4.0) for those that did not mention endorsement of the CONSORT statement although this was not a significant difference (*p*=0.064).

**CONCLUSIONS:**

Although there has been a significant improvement in the reporting of trials of operative intervention published in the surgical literature since 2004, items specific to the CONSORT-NPT extension remain underreported. Improved awareness of this important addition to the CONSORT statement throughout the surgical community and its endorsement by surgical journals will help to improve the reporting practice of trials of operative intervention.

The randomised controlled trial (RCT) represents the gold standard method for determining an association between treatment and outcome.^1^ As important as the quality of the trial is the quality of its reporting; without transparent reporting, adequate appraisal of a trial’s methodological quality and external validity is not possible. The Consolidated Standards of Reporting Trials (CONSORT) statement provides a minimum set of recommendations for the reporting of RCTs and is endorsed by many peer reviewed journals.^2^ Its endorsement is widely recognised to have improved the reporting quality of RCTs.^3^ However, since its introduction and update in 2001,^4^ numerous articles have identified ongoing reporting quality deficiencies in both the medical^3,5–7^ and surgical^8–10^ literature.

Trials of operative intervention carry inherent methodological challenges^11^ and so the CONSORT statement in this setting is not as useful as for trials of pharmacological intervention. Such challenges include accounting for variation in the recruiting and consenting practices of surgeons, difficulty in blinding patients and outcome assessors, the presence of confounding factors such as the surgeon’s technical ability, differing anaesthetic technique and the need to standardise operative technique between surgeons who have different training backgrounds. As a result, the emphasis of reporting is different than for trials of pharmacological intervention. The reporting of certain methodological features gains increased importance while other features are entirely unique to surgical trials.

The CONSORT statement for non-pharmacological treatment (CONSORT-NPT) provides criteria specific for such features, enabling assessment of the reporting quality of trials of operative (and other non-pharmacological) intervention while maintaining important reporting elements of the original CONSORT statement.^12^ Such items specific to the CONSORT-NPT checklist include the way in which interventions are standardised between surgeons, the assessment of a surgeon’s adherence to such standardisation, the volume of participants treated per surgeon and the surgeon’s experience with the intervention.

To date there has been no published analysis of the adherence of trials of operative intervention to the CONSORT-NPT statement or assessment of whether the CONSORTNPT extension has led to an improvement in the reporting quality of such trials. This study analyses the adherence of trials of operative intervention to the CONSORT-NPT statement at time periods before and after its publication in order to determine whether the CONSORT-NPT extension has contributed to an improvement in the reporting standard of trials of operative intervention.

## Methods

### Search strategy

Electronic searches of MEDLINE® and Embase™ were performed by a librarian at the John Radcliffe Hospital, Oxford. The following journals were searched: *British Journal of Surgery*, *Archives of Surgery*, the *European Journal of Cardio-Thoracic Surgery*, *European Urology* and *Journal of Bone and Joint Surgery* (British volume). These publications were chosen to represent high impact factor journals providing information from a range of surgical specialties. The *Annals of Surgery* was excluded as a preliminary search revealed that it published relatively few RCTs for the time periods studied. All included journals (with the exception of the *European Journal of Cardio-Thoracic Surgery*) have endorsed the CONSORT statement in their instructions to authors since 2007. No journal had mentioned the CONSORT-NPT statement as of February 2012 and the *European Journal of Cardio-Thoracic Surgery* had not mentioned the CONSORT or CONSORT-NPT statements.

**Figure 1:**
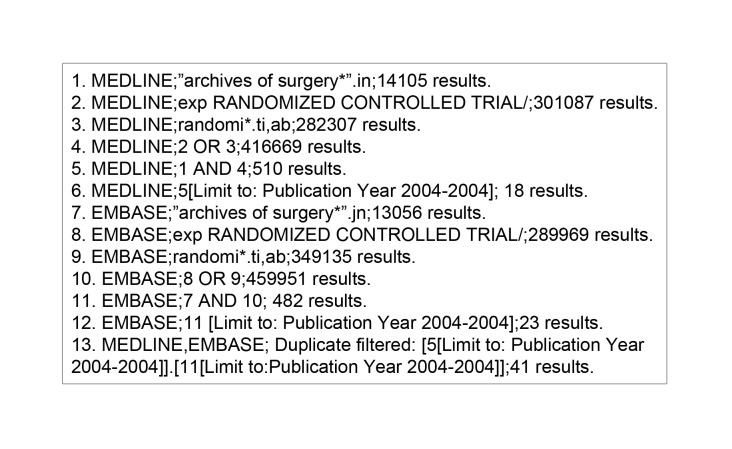
Search strategy for identification of randomised trials of operative intervention

The search was limited to two time periods: January to December 2004 (sufficiently long enough following the 2001 CONSORT statement revision^4^ that trialists should be aware of its publication) and January to December 2010 (similarly with regard to the publication of the CONSORT-NPT statement in 2008).^12^ The search history for an example journal can be seen in Figure 1. The search was repeated for each journal at each time point to ensure that all published RCTs within the search limits were retrieved. The NHS Evidence advanced search software (http://www.library.nhs.uk/) was used to perform electronic searches.

### Inclusion/exclusion criteria

RCTs were included if they reported the comparison of at least one non-pharmacological intervention. This included trials of surgical technique, surgical access, technology and instrument design as well as anaesthetic interventions. RCTs comparing solely pharmacological interventions were excluded. All journals included in this study publish their manuscripts in English.

Studies were included in chronological order until a sample size of ten trials per journal per time point had been reached. This limit was set based on labour constraints rather than performing a sample size calculation, which, following personal communication with the study’s lead statistician (DA), was felt to be unnecessary given that a defined hypothesis was not being tested.

Publications of retrospective, observational, experimental and animal studies were excluded as were trials reporting follow-up data of a previously published trial. Journals that endorse the CONSORT statement were defined as those that reference the statement in their instructions to authors or those journals that are referenced on the relevant CONSORT webpage (http://www.consort-statement.org/about-consort/consort-endorsement/consort-endorsers---journals/). Journals were not excluded on the basis of their lack of endorsement of the CONSORT statement.

All abstracts retrieved from database searching were reviewed for selection by two authors (AGW and RG). Studies in which it was not clear whether the inclusion criteria had been met were reviewed in full text and discrepancies between the two authors were resolved by discussion with the remaining authors.

### Data extraction

All publications were reviewed in full text by two authors (AGW and RG). From the CONSORT-NPT statement, a 30-point scoring system was devised (Table 1) giving equal weighting to CONSORT-NPT items and resulting in a score out of 30 for each trial. Two further items not included in the CONSORT-NPT statement (method of anaesthesia and sources of funding) were added as it was felt that these factors could be significant confounders in the design of trials of operative intervention such that their reporting was key. The reporting of the effect of clustering on sample size calculation and the estimate of the effect size and its precision were allocated their own point on the scoring system.

**Table 1:** The 30-point scoring system developed from the CONSORT-NPT and used to assess publications meeting the inclusion criteria

Paper section	CONSORT-NPT item	Description
Introduction	Background	Introduction and scientific background. Explanation of rationale for study, objectives and hypotheses
Methods	Eligibility (participants)	Participant eligibility criteria outlined clearly
	Eligibility (centres/surgeons)*	Outlining the eligibility criteria for centres involved in the trial (multicentre) and of those surgeons participating in the trial
	Study setting	Setting in which the intervention is administered stated clearly
	Intervention/control	Details of the operative intervention and control such that they could be performed by the reader, any changes required to the intervention or control for specific patients
	Intervention standardisation*	How operative techniques were standardised between participating surgeons
	Surgeons’ adherence*	Details of how operating surgeons’ adherence to standardised interventions or protocols was monitored
	Anaesthetic**	Type of anaesthesia used and the number of participants receiving different anaesthetic methods
	Outcome measures	Clearly defined primary and secondary outcome measures
	Sample size	Explanation of how sample size was determined
	Clustering (surgeons/centres)*	Explanation of how the effects of clustering in care providers was addressed
	Randomisation: sequence generation	Method used to generate the random allocation sequence
	Randomisation: allocation concealment	Method used to implement the allocation sequence (sealed envelopes, telephone etc)
	Implementation	Description of who generated the random sequence and assigned participants to their groups
	Degree of blinding	Description of whether participants, surgeons and/or outcome assessors were blinded
	Blinding method*	Description of the methods used to blind participants, surgeons or outcome assessors
	Statistical methods	Statistical methods used to compare groups for outcomes
Results	Participant flow	Flow of participants through each stage (diagram recommended but not a necessity) including the numbers of participants randomised, receiving each intervention, completing the study and analysed for each outcome
	Participant volume per centre/surgeon*	Documentation of the number of participants treated by each surgeon/centre
	Recruitment and follow-up dates	Dates during which participant recruitment was undertaken and when follow-up was arranged
	Demographics	Summary of baseline demographic data and clinical characteristics
	Experience*	Description of the centre or surgeon's experience with the technique including data on previous case volume, qualifications and expertise
	Analysis	Documentation of the numbers used in analysis (absolute numbers)
	Documentation of outcomes	Documentation of the findings for both the primary and secondary outcomes
	Effect size and precision	Documentation of estimated effect size and its precision (95% confidence intervals)
	Adverse events	Documentation of adverse events in the study group
Discussion	Interpretation	Interpretation of results including discussion regarding sources of bias, imprecision and shortcomings. If indicated, discussion of lack of blinding, unequal expertise of surgeons etc.
	Generalisability	Discussion regarding the external validity or otherwise of the study (in particular, the experience of the surgeons, type of centre and participant selection)
	Evidence	Discussion of the study results in the context of current evidence and opinion
Additional	Funding**	Description of sources of funding

* items with features specific to the CONSORT-NPT statement

** items added by the authors for the purpose of this study

Reporting of intention to treat was not included as an item in the scoring system as previous studies have provided evidence to suggest that this method of analysis is frequently misreported by trialists.^13^ Any addition or retraction of items from the original CONSORT-NPT checklist was done following discussion with all listed authors including a senior member of the CONSORT Group (DA). It has been noted that the CONSORT Group does not recommend the use of the checklist to provide a quality ‘score’.^14^ However, this approach gives a useful summary of overall reporting standard when comparing time periods to complement comparison of the reporting of specific items.

The data extracted by each author were compared and any discrepancies resolved with discussion between the authors and re-review of the publication. Any remaining discrepancies were discussed with a third author (MS) until a consensus had been reached and a final score obtained. Assessors were not blinded to the time period or journal in which the RCT was published.

### Outcome measures

The primary outcome measure was the difference in mean total modified CONSORT-NPT statement score between the 2004 and 2010 time periods. The secondary outcome measures were both percentage of adherence to each CONSORT-NPT item and the difference in mean total modified CONSORT-NPT statement score between those journals endorsing the CONSORT-NPT statement and those not doing so.

### Statistical analysis

Normally distributed means were compared using Student’s t-test and non-normally distributed data with the Mann–Whitney U test. Normality was determined by visualisation of histograms and *p*-values of <0.05 were considered statistically significant.

## Results

### Study flow, demographics and CONSORT-NPT endorsement

**Figure 2:**
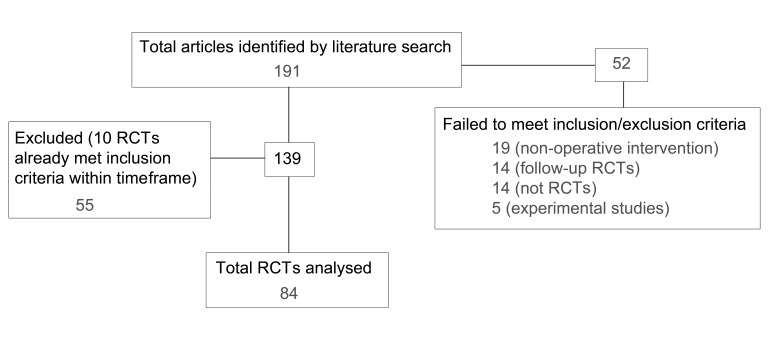
Flowchart of study showing included and excluded randomised controlled trials (RCTs)

The study flow can be seen in Figure 2. A total of 191 RCTs were identified with 81 and 110 published in 2004 and 2010 respectively. Of these, 55 publications were excluded because the maximum number of RCTs had already been reviewed for specific journals within specific time points. The demographic details for publications at each time point were similar (Table 2).

**Table 2:** Demographics of included studies

		Publication year	
		**2004**	**2010**
**Number of trials**		44	40
**Participants**		7,082 (58%)	5,187 (42%)
**Location**	Europe	30 (68%)	28 (70%)
	North America	6 (14%)	5 (13%)
	Asia	4 (9%)	5 (13%)
	Oceania	3 (7%)	2 (5%)
	Africa	1 (2%)	0 (0%)
	South America	0 (0%)	0 (0%)
**Specialty**	Cardiothoracics	9 (20%)	10 (25%)
	Orthopaedics	10 (23%)	8 (20%)
	Urology	5 (11%)	8 (20%)
	General surgery	20 (45%)	14 (35%)
**Organisation**	Single centre	34 (77%)	31 (78%)
	Multi centre	10 (23%)	9 (23%)
**Design**	Parallel	36 (82%)	36 (90%)
	Multiple arms	8 (18%)	4 (10%)
	Intervention	41 (93%)	39 (98%)
	Diagnostic	3 (7%)	1 (3%)
**Journal**	Endorses CONSORT	35 (80%)	30 (75%)

### Comparison of mean CONSORT-NPT score

The mean CONSORT-NPT score was 15.2 (standard deviation [SD]: 3.8) for RCTs published in 2004 and 19.1 (SD: 4.1) for those published in 2010. The improvement in mean CONSORT-NPT score from 2004 to 2010 was 3.95 points (95% confidence interval: 3.61–4.29, *p*<0.001). There was considerable variation in the reporting of individual CONSORT-NPT items with several items being underreported at both time points (Table 3). No single trial scored 100%.

**Table 3:** The number of trials that reported each CONSORT-NPT item for articles published in 2004 and 2010

Paper section	CONSORT-NPT item	Trials reporting item 2004	2010	*p*-value
Introduction	Background	32 (73%)	29 (73%)	1.0
Methods	Eligibility (participants)	32 (73%)	36 (90%)	**0.045**
	Eligibility (centres/surgeons)*	4 (9%)	11 (27%)	**0.029**
	Study setting	17 (39%)	23 (58%)	0.086
	Intervention/control	40 (91%)	38 (95%)	0.47
	Intervention standardisation*	18 (41%)	17 (43%)	0.88
	Surgeons’ adherence*	2 (5%)	3 (8%)	0.57
	Anaesthetic**	17 (39%)	16 (40%)	0.9
	Outcome measures	36 (82%)	32 (80%)	0.83
	Sample size	20 (45%)	32 (80%)	**<0.001**
	Clustering (surgeons/centres)*	0 (0%)	1 (3%)	0.29
	Randomisation: sequence generation	23 (52%)	28 (70%)	0.064
	Randomisation: allocation concealment	21 (48%)	29 (73%)	**0.022**
	Implementation	11 (25%)	21 (53%)	**0.010**
	Degree of blinding	20 (45%)	21 (53%)	0.52
	Blinding method*	19 (43%)	19 (48%)	0.69
	Statistical methods	42 (95%)	40 (100%)	0.34
Results	Participant flow	18 (41%)	32 (80%)	**<0.001**
	Participant volume per centre/surgeon*	9 (20%)	17 (43%)	0.054
	Recruitment and follow-up dates	38 (86%)	39 (98%)	0.067
	Demographics	36 (82%)	40 (100%)	**0.003**
	Experience*	5 (11%)	15 (38%)	**0.005**
	Analysis	42 (95%)	39 (98%)	0.62
	Documentation of outcomes	42 (95%)	36 (90%)	0.34
	Effect size and precision	9 (20%)	14 (35%)	0.14
	Adverse events	35 (80%)	35 (88%)	0.33
Discussion	Interpretation	15 (34%)	28 (70%)	**0.003**
	Generalisability	12 (27%)	18 (45%)	0.2
	Evidence	37 (84%)	38 (95%)	0.19
Additional	Funding**	20 (45%)	28 (70%)	**0.012**

*items with features specific to the CONSORT-NPT statement

**items added by the authors for the purpose of this study

### Adherence to the CONSORT-NPT statement

Regarding methodological issues, for RCTs published in 2010 there was a significant increase in the reporting of surgeon’s/centre’s eligibility criteria, sample size calculation, method for random sequence generation and allocation concealment (Table 3). For the results section, a significantly higher percentage of RCTs published in 2010 included a flow diagram or at least sufficient information to determine participant flow. There was also a significant improvement in the reporting of the number of participants treated per surgeon or centre, the study population’s demographics and the experience of the surgeon or centre with the intervention technique. Finally, a significantly higher percentage of studies published in 2010 highlighted potential areas of bias.

**Figure 3:**
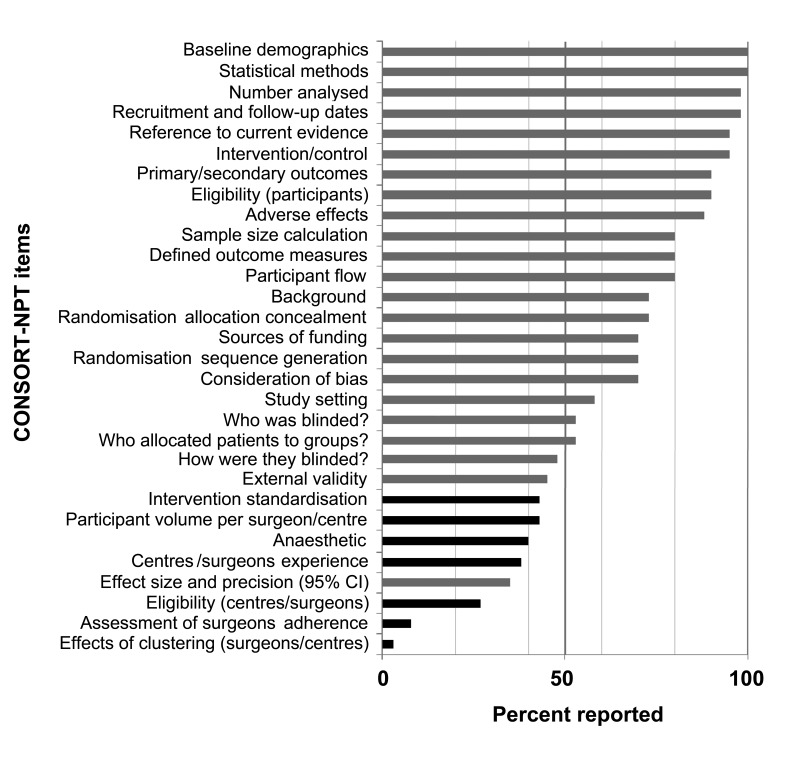
Percentage of adherence to the CONSORT-NPT statement for trials published in 2010

Methodological items reported by <50% of RCTs in 2010 include the eligibility criteria on which surgeons or centres were selected (27%), the type of anaesthetic used (40%), how interventions were standardised between surgeons (43%), the methods used to monitor surgeons’ adherence to the intervention or comparator techniques (8%), the effect that clustering has on the sample size calculation (3%) and the method of blinding participants or outcome assessors (48%). For the reporting of results, the number of participants treated by each surgeon or centre (43%), the surgeon’s/centre’s experience (38%) and the confidence of the effect estimate (35%) were all poorly reported (Fig 3).

### CONSORT-NPT score and journal practice

The mean CONSORT–NPT score (both time points) for those studies published in CONSORT endorsing journals (mean: 17.5, SD: 4.5) was higher than that for the *European Journal of Cardio-Thoracic Surgery* (mean: 15.6, SD: 4.0). However, this did not reach statistical significance (*p*=0.064). When this was analysed per time period there was also no significant difference.

## Discussion

Trials involving operative rather than pharmacological intervention bring inherent methodological challenges. Failure to overcome such challenges in the conduct of a trial is likely to lead to considerable bias, potentially invalidating the results and limiting their interpretation. Adherence to the CONSORT statement enables trial authors to maintain a transparent system of reporting so that the reader can draw considered conclusions from the trial findings. Previous studies have found that trials published in the surgical literature are lacking in their adherence to the CONSORT statement.^8–10^

Balasubramanian *et al* found that trials published in high impact surgical journals reported only 69% of CONSORT items.^9^ Similarly, studies analysing the reporting quality of publications in spinal^15^ and cardiothoracic^16^ surgery found on average 65% and 66% of CONSORT items were reported respectively while the figure for urological trials was lower still at 52%.^8^

All such studies acknowledge the difficulties in performing surgical trials. However, all determined CONSORT statement adherence rather than CONSORT-NPT adherence and included trials of pharmacological as well as operative intervention in their assessment. This makes them less specific for the analysis of operative trials and the reporting of methodological features, which make such trial design difficult. Furthermore, no previous study has analysed reporting adequacy in trials of operative intervention at two time points such that until this time any change in reporting standards could not be quantified.

It is recognised that in conducting this study the analysis is limited to four surgical specialties and that although data extraction was not performed in a blinded fashion, consensus was reached between multiple authors when scoring trials. The high quality of the search strategy and well defined inclusion/exclusion criteria enabled the analysis solely of trials of surgical intervention at two time points separated by the introduction of the CONSORT-NPT statement.

Equal weighting was given to all items in the CONSORT-NPT statement to create an overall score although some items may in fact assume greater importance than others. Nevertheless, presentation of the figures for the reporting of each item individually (Table 2) enables a clearer understanding of the items most frequently underreported. Finally, although the demographics of the included trials differed little between the two time periods studied (Table 2), it is recognised that improvements seen in the CONSORT-NPT score in the period studied could result from secular trends, in particular an improved awareness of an evidence-based approach in the surgical community or stricter ethical regulations imposed on trials rather than publication of the CONSORT-NPT statement alone.

Importantly, the significant improvement in reporting from 2004 to 2010 resulted from improved reporting of items such as sample size calculation, allocation concealment and participant flow (all items found in the original CONSORT statement). Comparison with prior estimates of reporting practice supports this improvement. For example, previous estimates for the number of trials in the surgical literature reporting sample size calculations were 20% (2003)^16^ and 44% (2008)^15^ compared with 80% (2010) here. Similarly, the percentage of trials reporting study flow was estimated at 51% (2000–2003)^8^ and 52% (2008),^15^ and documented as 80% (2010) here. This trend is repeated for reporting of participant blinding, random sequence generation and allocation concealment among other items.

## Conclusions

Although CONSORT items improved significantly, there was little improvement in CONSORT-NPT specific items, all of which were reported in less than 50% of trials in 2010 (Fig 3). While journals give specific instructions to authors regarding the use of the CONSORT statement, no mention of the CONSORT-NPT statement was found in the journals included in this study. Peer reviewed journals’ instructions to authors is likely to have played a large part in improving the awareness of reporting standards throughout surgical academia by insisting on adherence to the CONSORT guidance. The evidence presented here strongly suggests that journals publishing trials of operative intervention should pay equal attention to the CONSORT-NPT statement with the aim being to improve both authors’ and reviewers’ awareness of this CONSORT extension. This will, in turn, help to improve the quality of reporting of methodological issues specific to such trials, enabling clearer interpretation of their outcomes.

## References

[1] Abel U, Koch A. The role of randomization in clinical studies: myths and beliefs. J Clin Epidemiol 1999; 52: 487–497 10408986 10.1016/s0895-4356(99)00041-4

[2] Begg C, Cho M, Eastwood S*et al.* Improving the quality of reporting of randomized controlled trials. The CONSORT statement. JAMA 1996; 276: 637–639 8773637 10.1001/jama.276.8.637

[3] Moher D, Jones A, Lepage L. Use of the CONSORT statement and quality of reports of randomized trials: a comparative before-and-after evaluation. JAMA 2001; 285: 1,992–1,995 10.1001/jama.285.15.1992 11308436

[4] Moher D, Schulz KF, Altman DG. The CONSORT statement: revised recommendations for improving the quality of reports of parallel-group randomised trials. Lancet 2001; 357: 1,191–1,194 11323066

[5] Devereaux PJ, Manns BJ, Ghali WA*et al.* The reporting of methodological factors in randomized controlled trials and the association with a journal policy to promote adherence to the Consolidated Standards of Reporting Trials (CONSORT) checklist. Control Clin Trials 2002; 23: 380–388 12161081 10.1016/s0197-2456(02)00214-3

[6] Haidich AB, Birtsou C, Dardavessis T*et al.* The quality of safety reporting in trials is still suboptimal: survey of major general medical journals. J Clin Epidemiol 2011; 64: 124–135 21172601 10.1016/j.jclinepi.2010.03.005

[7] Mills EJ, Wu P, Gagnier J, Devereaux PJ. The quality of randomized trial reporting in leading medical journals since the revised CONSORT statement. Contemp Clin Trials 2005; 26: 480–487 16054580 10.1016/j.cct.2005.02.008

[8] Agha R, Cooper D, Muir G. The reporting quality of randomised controlled trials in surgery: a systematic review. Int J Surg 2007; 5: 413–422 18029237 10.1016/j.ijsu.2007.06.002

[9] Balasubramanian SP, Wiener M, Alshameeri Z*et al.* Standards of reporting of randomized controlled trials in general surgery: can we do better? Ann Surg 2006; 244: 663–667 17060756 10.1097/01.sla.0000217640.11224.05 PMC1856614

[10] Sinha S, Sinha S, Ashby E*et al.* Quality of reporting in randomized trials published in high-quality surgical journals. J Am Coll Surg 2009; 209: 565–571.e1 19854395 10.1016/j.jamcollsurg.2009.07.019

[11] McCulloch P, Taylor I, Sasako M*et al.* Randomised trials in surgery: problems and possible solutions. BMJ 2002; 324: 1,448–1,451 12065273 10.1136/bmj.324.7351.1448 PMC1123389

[12] Boutron I, Moher D, Altman DG*et al.* Extending the CONSORT statement to randomized trials of nonpharmacologic treatment: explanation and elaboration. Ann Intern Med 2008; 148: 295–309 18283207 10.7326/0003-4819-148-4-200802190-00008

[13] Gravel J, Opatrny L, Shapiro S. The intention-to-treat approach in randomized controlled trials: are authors saying what they do and doing what they say? Clin Trials 2007; 4: 350–356 17848496 10.1177/1740774507081223

[14] Schulz KF, Altman DG, Moher D. CONSORT 2010 statement: updated guidelines for reporting parallel group randomised trials. BMJ 2010; 340: c332 20332509 10.1136/bmj.c332 PMC2844940

[15] Naunheim MR, Walcott BP, Nahed BV*et al.* The quality of randomized controlled trial reporting in spine literature. Spine 2011; 36: 1,326–1,330 21224750 10.1097/BRS.0b013e3181f2aef0

[16] Tiruvoipati R, Balasubramanian SP, Atturu G*et al.* Improving the quality of reporting randomized controlled trials in cardiothoracic surgery: the way forward. J Thorac Cardiovasc Surg 2006; 132: 233–240 16872940 10.1016/j.jtcvs.2005.10.056

